# The Impact of Aquatic Exercise on Walking Ability, Quality of Life, and Enjoyment in Children with Cerebral Palsy: A Systematic Review

**DOI:** 10.3390/pediatric17010002

**Published:** 2025-01-02

**Authors:** Miki Nakatani, Yuji Kanejima, Kodai Ishihara, Hanhwa Koo, Kazuhiro P. Izawa

**Affiliations:** 1 Department of Health Science, Faculty of Medicine, Kobe University, Kobe 654-0142, Japan; 2 Department of Public Health, Graduate School of Health Sciences, Kobe University, Kobe 654-0142, Japan; 3 Cardiovascular Stroke Renal Project (CRP), Kobe 654-0142, Japan; 4 Department of Rehabilitation, Kobe City Medical Center General Hospital, Kobe 650-0047, Japan; 5 Department of Physical Therapy, Faculty of Nursing and Rehabilitation, Konan Women’s University, Kobe 658-0001, Japan

**Keywords:** cerebral palsy, child, aquatic therapy, systematic review

## Abstract

Background/Objectives: Aquatic exercise is attracting attention as a method of rehabilitation for children with cerebral palsy (CP). The purpose of this systematic review was to evaluate whether aquatic exercise for children with CP improves their walking ability and quality of life (QOL) and is enjoyable for them. Methods: A literature search was conducted on 2 August 2024 using three databases: PubMed, Web of Science, and the Cochrane Central Register of Controlled Trials through Evidence-Based Medicine Reviews. Studies included in the review focused on aquatic exercise interventions for children with CP, with outcomes related to walking ability, QOL, or enjoyment. Studies that did not isolate the effects of aquatic exercise (except when combined with conventional interventions) were excluded. Two reviewers independently conducted screening and risk of bias assessments. Results: Seven studies involving 94 participants in total were included in the review. Three of these studies had a control group. All four studies reported improvements in walking ability, including walking endurance and efficiency. One of two studies showed improvement in health-related QOL (HRQOL) compared to the control group, whereas the other did not show significant differences between groups. All three studies that assessed enjoyment reported high levels of enjoyment of aquatic exercise. However, all studies were at risk of bias. Conclusions: The reviewed studies suggest that aquatic exercise for children with CP may be enjoyable and may improve walking ability. Further research is needed to accurately assess the effects of aquatic exercise and compare it to other interventions.

## 1. Introduction

Cerebral palsy (CP) describes a group of permanent disorders of the development of movement and posture that cause activity limitation and are attributed to non-progressive disturbances occurring in the developing fetal or infant brain [1]. The prevalence of CP is 2.11 per 1000 live births worldwide, and CP is the most common movement disorder in children [2]. Rehabilitation is essential for children with CP because of its effects on postural and motor development.

A common therapeutic goal of rehabilitation for children with CP is to improve their mobility and walking ability [3]. Reduced walking speed, endurance, and efficiency may limit the ability of children with CP to move with their peers [4]. Therefore, improved walking ability may reduce limitations in participation. In addition, motor disorders in CP often coincide with sensory, cognitive, and behavioral disturbances, as well as epilepsy and musculoskeletal complications [1]. So, many children with CP have non-movement impairments that may affect their quality of life (QOL) [5]. In one study, children with CP self-reported a significantly lower health-related QOL (HRQOL) than healthy children [6]. Improved QOL helps children with CP lead richer lives. Furthermore, in a study of young adults with childhood-onset physical disabilities, fun and social contacts were mentioned as facilitators of engaging in physical activity [7], thus indicating that enjoyment is an important aspect of physical activity and rehabilitation for children with disabilities.

Aquatic exercise is attracting attention as a method of rehabilitation for children with CP that offers benefit to children with significant movement limitations for whom participation in land-based exercise may be limited [8]. Aquatic exercise appeals to children with CP because of the unique quality of buoyancy in water that reduces joint loading and impact and decreases the negative influences of poor balance and poor postural control [8]. Previous systematic reviews suggested that aquatic intervention for children with CP may improve motor function as represented by gross motor function [9,10,11]. The Gross Motor Function Measure, an index used to assess gross motor function in children with CP, was shown to correlate with gait parameters [12]. Thus, aquatic exercise may improve not only gross motor function but also walking ability, including walking speed, endurance, and efficiency.

There are few systematic reviews that include current studies on how aquatic exercise affects walking ability, QOL, and enjoyment for children with CP. We hypothesized that aquatic exercise would improve walking ability and QOL and be enjoyable for children. Thus, the purpose of this systematic review was to evaluate whether aquatic exercise for children with CP improves their walking ability and QOL and is enjoyable for them.

## 2. Materials and Methods

This review was conducted in accordance with the Preferred Reporting Items for Systematic Reviews and Meta-Analyses (PRISMA) statement [13]. The study was registered in the OSF registries (https://doi.org/10.17605/OSF.IO/UD964, accessed on 14 December 2024).

### 2.1. Eligibility Criteria

Studies meeting the following inclusion criteria were included: (1) participants were children with CP, (2) participants aged 0–19 years old, (3) aquatic exercise intervention was performed, (4) the study was written in English, and (5) the study was published after 2006. We excluded studies that met the following exclusion criteria: (1) interventions did not show the effects of aquatic exercise alone (concomitant use with conventional therapy was not excluded), (2) neither walking ability (excluding gross motor functions only), QOL, nor enjoyment was included in the outcomes, and (3) no statistical analysis was described (except for indicators related to enjoyment).

### 2.2. Search Strategy

**Figure 1:**
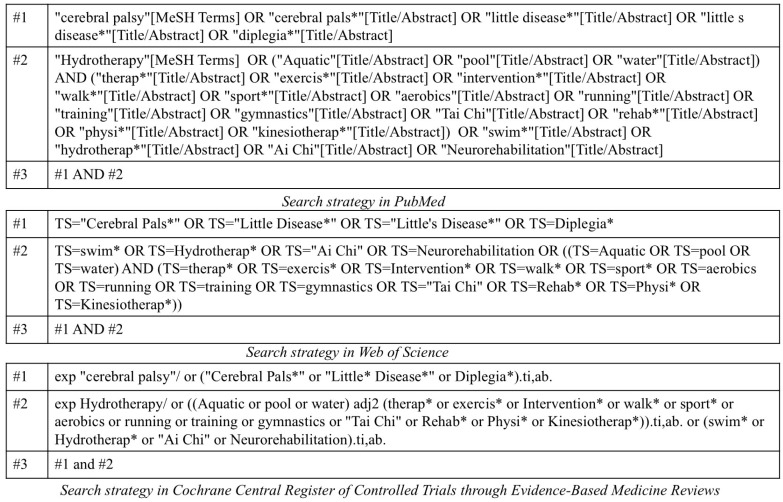
Search strategy for the present study. PubMed, Web of Science, and Cochrane Central Register of Controlled Trials through Evidence-Based Medicine Reviews were used for the search. In this search query, the asterisk (*) is used as a wildcard character to represent any number of characters. This allows for the inclusion of various forms of a word.

The search for articles was conducted by one researcher (M.N.) on 2 August 2024. Three databases (PubMed, Web of Science, and Cochrane Central Register of Controlled Trials through Evidence-Based Medicine Reviews) were used for the search. The search strategy was reviewed by a librarian. Search strategies were developed using search terms related to “cerebral palsy”, “aquatic”, and “exercise” (Figure 1).

### 2.3. Study Selection

All retrieved articles were managed with Rayyan https://www.rayyan.ai/ (accessed on 2 August 2024), and duplicate articles were removed [14]. The titles and abstracts were read, and articles that did not clearly meet the selection criteria were excluded. Articles for which full text was not available were also excluded. Finally, the full text of the selected articles was read, and the reasons for exclusion were clearly stated. This was performed independently by two researchers (M.N. and H.K.). Disagreements were discussed with a third reviewer (K.I.).

### 2.4. Data Extraction

The following information was extracted from the articles by reviewer M.N.: author and publication year, study type, sample size, age, Gross Motor Function Classification System (GMFCS) level, outcomes (walking ability, QOL, and enjoyment), measurements, intervention, and results.

### 2.5. Risk of Bias Assessment

**Figure A1:**
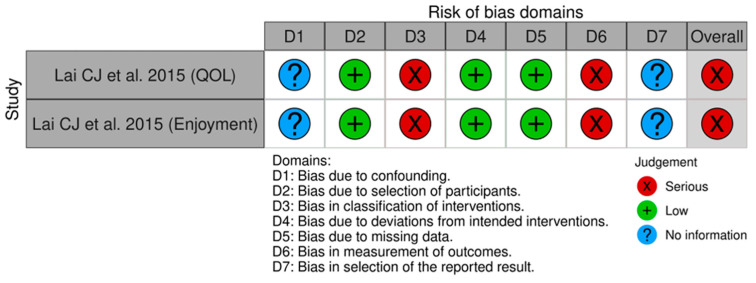
Quality assessment by ROBINS-I (Risk Of Bias In Non-Randomized Studies—of Interventions) [19].

Two reviewers (M.N. and H.K.) independently assessed the risk of bias of the studies. Disagreements were discussed with a third reviewer (Y.K.). We used the Revised Cochrane risk-of-bias tool for randomized trials (RoB2) to assess the methodological quality of the randomized controlled trials (RCTs) [15]. The RoB2 tool was used to evaluate each outcome, with an overall rating of “low risk”, “some concern”, or “high risk” according to signaling questions for the five domains. Risk Of Bias In Non-Randomized Studies—of Interventions (ROBINS-I) was used for non-RCTs [16]. The ROBINS-I tool gave an overall rating of “Serious”, “Low”, or “No information” according to the signaling questions in each of the seven domains and was used to evaluate each outcome. The table in Figure A1 was created using the Risk-of-bias VISualization tool (robvis) [17]. For the non-controlled studies, we used the Quality Assessment Tool for Before–After (Pre–Post) Studies with no Control Group [18]. This tool was used to assess each outcome, with 12 questions answered with “Yes”, “No”, “cannot determine”, “not applicable”, or “not reported”. The tool was not designed to provide a list of the factors comprising a numeric score, so the total number of “Yes” decisions (indicating low risk) was noted.

## 3. Results

### 3.1. Study Selection

**Figure 2:**
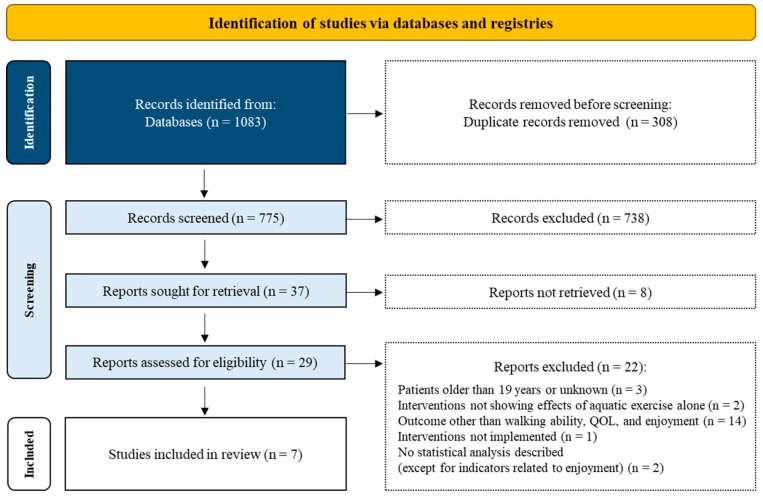
Flow diagram of the present study. Flow diagram showing the flow of study selection in the present study. Articles excluded in the second screening were noted with the reason for exclusion, and finally, seven articles were included.

A search of the databases yielded 1083 articles in total, of which 363 articles were obtained from PubMed, 633 from Web of Science, and 87 from the Cochrane Central Register of Controlled Trials through Evidence-Based Medicine Reviews. After identifying and removing 308 duplicate articles, the titles and abstracts of 775 articles were screened, and 738 were determined to be irrelevant to the present study. Among the 37 relevant articles, the full text could not be obtained for 8 of them. Thus, after reading the full text of the remaining 29 articles, 7 were finally included in the study. The reasons for exclusion at the full-text screening stage were as follows: patients older than 19 years or age unknown (n = 3), interventions not showing effects of aquatic exercise alone (n = 2), outcomes other than walking ability, QOL, and enjoyment (n = 14), interventions not implemented (n = 1), and no statistical analysis described except for indicators related to enjoyment (n = 2) (Figure 2).

### 3.2. Study Characteristics

The articles included in this review are two RCTs, one quasi-experimental study, and four studies without control groups published between 2009 and 2024. The characteristics of the included studies are summarized in Table 1. Sample sizes ranged from 1 to 32, participant ages ranged from 4 to 17 years old, and GMFCS levels ranged from I to V. Excluding one study with one female participant only, the percentage of males ranged from 50% to 66.7%. In most studies, interventions were conducted by physical therapists. The duration of the aquatic exercise intervention was 40–60 min per session over one to five sessions per week. Intervention periods ranged from 6 weeks to 8 months. In most studies, conventional treatment or rehabilitation was continued or unknown. Of the three studies with a control group, two provided only conventional treatment or rehabilitation in the control group and one used a land-based exercise program in the control group.

In two studies, the aquatic exercise programs were based on the Halliwick concept [19,20], which has three levels of learning: (1) mental adjustment, (2) balance control, and (3) movement [11]. In one study, the swimming program goals were to improve independence in the water and to learn or improve swimming strokes, and the interventions included swimming stroke practice, free play, races, and other games [21]. Aquatic exercise in the remaining four studies all included aerobic exercise [22,23,24,25], and two of them also included strengthening exercises in water [23,24]. Aerobic exercise included walking, running, jumping, hopping, creeping, kicking, swimming, step climbing, and treading water. Strengthening exercises included those for the lower extremities, trunk, and upper extremities.

### 3.3. Effects of Aquatic Exercise on Walking Ability

Four studies measured walking ability. The 1-min walk test (1MWT), 6-min walk test (6MWT), and Modified Energy Expenditure Index (MEEI) were used as the indicators. GMFCS levels in children with CP evaluated for walking ability ranged from I to III.

Two studies used the 1MWT, with improvements in walking ability observed in both studies. The study by Declerck et al. [21] showed a significantly greater increase in walking ability in the intervention group after the 10-week swimming program than in the control group, with an average increase of 11.6 m in walking distance (*p* = 0.043). There were no significant differences between groups for the changes occurring over the 15-week period, which included a 5-week follow-up. However, the swimming group increased their walking distance by 18.9 m compared to 4.9 m in the control group. Twenty weeks after completing the swimming program, the swimming intervention group retained a significant increase from baseline.

According to the study by Fatorehchy et al. [25], significant changes were found between initial and final testing (*p* = 0.041). The mean values of the 1MWT at initial and final measurements were, respectively, 19.16 m and 20.66 m. However, this change did not reach the minimum clinically important difference in the 1MWT by GMFCS level [26].

Two non-controlled studies measured walking ability with the 6MWT, and both showed improved walking endurance. In the single-subject design study conducted by Retarekar et al. [22], improvements in walking endurance were observed that showed an increase of 27.1% in the distance walked in 6 min from the mean of the baseline phase (232.94 m) to the end of the intervention phase (296 m). In the 3-month follow-up phase, the participant’s speed and distance for the 6MWT progressively decreased over time, returning to baseline values.

The study by Fragala-Pinkham et al. [23] showed a stable baseline period with two baseline measurements and significant improvement from the two baseline measurements to post intervention for walking endurance (*p* = 0.004, 0.001). The respective mean 6-min walking distances were 340.8 m and 360.6 m at baseline measurements 1 and 2 and 424.3 m and 384.5 m post intervention and at the 1-month follow-up. It was suggested that the improvement in walking distance was not strong enough to be reliably maintained at the one-month follow-up.

One study measured the MEEI. In the single-subject design study conducted by Retarekar et al. [22], a significant decrease in MEEI from baseline to post intervention was observed. The decrease in MEEI suggests improved walking efficiency. Variability in MEEI scores was observed, and not all data points were significant in the intervention phase. The mean MEEI was lower in the intervention phase (3.12 beats/m) compared with that of the baseline (4.04 beats/m) and follow-up phases (3.61 beats/m). In the follow-up phase, the participant’s energy expenditure increased, indicating a decrease in walking efficiency.

### 3.4. Effects of Aquatic Exercise on QOL

Two studies evaluated HRQOL. One study showed greater improvement in HRQOL in the intervention group compared with the control group.

Adar et al. [24] used the Pediatric Quality of Life Inventory (PedsQL)-CP Module to assess HRQOL. They reported higher improvements in most subparts of the child self-report PedsQL, and the parent proxy report PedsQL, in the aquatic exercise group compared to the land-based exercise group. In the study, only the aquatic exercise group showed significant improvements in the daily activity, school activity, pain and injury, and eating activity subparts of the child self-report PedsQL, and in the daily activity, school activity, and fatigue subparts of the parent proxy report PedsQL. Significant improvements were noted in both groups in the movement and balance subpart of the child self-report PedsQL, and in the movement and balance and pain and injury subparts of the parent proxy report PedsQL after treatment.

The study by Lai et al. [19] reported no differences in HRQOL between the two groups in the subpart or total scores on the Cerebral Palsy Quality-of-Life–parent proxy scale.

### 3.5. Effects of Aquatic Exercise on Enjoyment

Three studies evaluated enjoyment, but only one study analyzed group differences.

In the study by Lai et al. [19], the intervention group had significantly higher post-intervention Physical Activity Enjoyment Scale scores than the control group (*p* = 0.015). In the study by Ogonowska-Slodownik et al. [20], aquatic therapy was conducted as part of the school program. An original questionnaire was used to assess the degree of satisfaction with the program. All participants declared that they enjoyed being in the swimming pool and found the classes ‘fun and cool.’ The students also selected swimming pool classes as their favorite among all the classes provided by the special education school. In the study by Declerck et al. [21], participants rated their perceived level of enjoyment of the swimming sessions on a 5-point Likert scale using smiley faces and labels. All individuals but one indicated that they enjoyed the swimming sessions “very much.” One child indicated that the sessions were enjoyed “a little bit”.

### 3.6. Risk of Bias in the Studies

**Figure 3:**
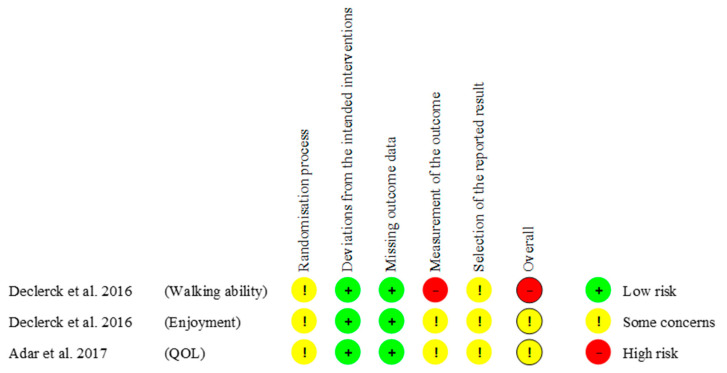
Quality assessment by Revised Cochrane risk-of-bias tool for randomized trials (RoB2). This figure shows the results of assessment of the risk of bias in the randomized controlled trials using the RoB2 tool. Evaluations were made for the five domains with information, and overall ratings were given. Assessments made for each outcome showed one with high risk and two with some concerns. QOL: quality of life [21,24].

All RCT studies had a low risk of bias for deviations from the intended interventions and missing outcome data. One RCT had a high risk of bias for measurement of the outcome for the outcome of walking ability (Figure 3). In addition, one non-RCT had a higher risk of bias in the classification of interventions and bias in the measurement of outcomes (Figure A1). All of the non-controlled studies were at risk of bias on some questions because they did not specify the timing or location of recruitment, or participants were not considered representative of the target population for the test/service/intervention in the general or clinical population of interest (Table A1).

## 4. Discussion

### 4.1. Summary of This Study

Focusing on aquatic exercise interventions for children with CP, the effects on walking ability, QOL, and enjoyment were systematically reviewed. Aquatic exercise interventions included interventions based on the Halliwick concept, aerobic exercise in water (such as walking, running, and jumping), and strengthening exercises for the lower extremities, trunk, and upper extremities. All studies showed improvements in walking ability in between-group or before/after comparisons, one of two studies showed improvements in HRQOL in between-group comparisons, and all studies reported high levels of enjoyment.

### 4.2. Effects of Aquatic Exercise on Walking Ability

The results of this study indicate that aquatic exercise has the potential to improve walking ability, such as walking endurance and efficiency, in children with CP. All studies were conducted in children at GMFCS levels I–III, so the impact on more severely affected children is unknown.

In a previous systematic review, many studies showed that aquatic interventions for children with CP improved gross motor function [9,10,11]. The present review showed a similar trend to these studies for walking ability, including walking endurance and efficiency. Aquatic exercise reduces the load and impact on joints and may allow a child to engage more easily in intensified strength and/or aerobic activity than does land-based exercise [8]. In addition, the viscosity of water creates resistance to movement, which can be used to strengthen muscles [27]. Patients can control reinforcing activities within their comfort zone [27]. The study by Fatorehchy et al. showed that aquatic exercise improved balance capacity, which may have contributed to improved walking ability [25]. Because there was only one study with a control group and the risk of bias was high, it cannot be determined that aquatic exercise improves walking ability compared to no aquatic exercise, but the possibility exists. Decreased walking ability can cause limitations in activity and participation for children with CP [4,28]. Therefore, improving walking ability through aquatic exercise may reduce these limitations.

### 4.3. Effects of Aquatic Exercise on QOL

In the studies showing improved HRQOL compared to controls, HRQOL was measured by child self-report and parent proxy report. Possible reasons for the improved HRQOL scores of the children with CP in the aquatic exercise group may be that exercise in the water increases confidence and reduces resistance to a difficult task [29]. This may have affected their daily and school activities.

However, the one study that did not show an improvement in HRQOL compared to the control group was measured by parent proxy report, which may not have accurately reflected changes in their children’s HRQOL. It may be better to include child self-reports to detect changes in HRQOL. The authors of that study stated that the small number of patients and the short duration of the intervention may also have been responsible for the lack of improvement in HRQOL [19].

Comparing the two studies, differences in intervention frequency may also have affected outcome results. One systematic review suggested that an aquatic program with an optimal duration of two months provided for approximately one hour/session at a frequency of more than twice a week has the capability to provide children with CP with improvements in motor functions [10]. The one study that did not show improvement in HRQOL compared to the control group provided a one-hour session twice a week over a 12-week aquatic exercise intervention, but more frequent interventions and more sessions may be needed to observe improvements in HRQOL.

### 4.4. Effects of Aquatic Exercise on Enjoyment

The included studies all suggested that children may be motivated to exercise in the water. Children with GMFCS levels I–V were included in the studies using enjoyment as an outcome. It thus appears that aquatic exercise may also have the potential to be enjoyable for children with varying degrees of motor disability.

Studies with adults have shown that aquatic environments and aquatic exercise reduce anxiety and increase well-being [30,31]. In addition, aquatic exercise may bring a sense of well-being to children and may lead to enjoyment because they can move more freely than on land. The enjoyment of aquatic exercise may motivate children to engage in it, thus contributing to improved motor function.

### 4.5. Limitations

This review has several limitations. First, the search was limited to three databases and only included published articles. Second, we restricted our search to articles written in English, which may have reduced the number of included studies. Third, a meta-analysis could not be conducted due to the small number of included articles, the variety of measures assessed for each outcome, and differing study designs. As a result, it is not possible to determine a causal relationship, and caution should be exercised when applying the results to clinical practice. Fourth, non-controlled studies were included due to the limited number of studies on this topic, making it difficult to compare outcomes with no aquatic exercise or other treatments. Finally, some included studies had a high risk of bias, necessitating careful interpretation of the results. The risk of bias was high for the outcome measurements, partly because children with CP were targeted. Therefore, the reliability of the results of each included study may be low. All non-controlled studies also exhibited bias in some domains. It should be noted that there are few studies on this topic, and this review also included case studies. This study reveals that there are few studies on this topic with sample sizes and blinding. Potential bias should be considered. To increase the evidence for aquatic exercise for children with CP in the future, higher-quality studies that include diverse populations and settings are needed. We suggest more randomized controlled trials (RCTs) or multicenter trials on this topic.

## 5. Conclusions

Aquatic exercise for children with CP may improve their walking ability, including endurance and efficiency, and may be enjoyable for them. Further studies are needed to accurately assess the effects of aquatic exercise, including comparisons with other therapies and studies with more participants.

## Figures and Tables

**Table 1:** Summary of included studies.

Author (Year)	Study Type	Sample	Outcome	Measurement	Intervention	Results
Lai et al. (2015) [19]	Quasi-experimental study	Size: n = 24 Age: 4 to 12 years GMFCS: I to IV	HRQOL, Enjoyment	Cerebral palsy quality-of-life–parent proxy scale, Physical activity enjoyment scale	60 min, twice a week, for 12 weeks. 5–10 min of warm up and stretching, 40 min of pool exercises, 5–10 min of exercises to cool down. The program was designed based on the Halliwick concept and comprised both aerobic and anaerobic training.	HRQOL: Did not differ between the 2 groups. Enjoyment: The intervention group scored significantly higher than the control group (*p* = 0.015).
Ogonowska-Slodownik et al. (2024) [20]	Single group study	Size: n = 9 Age: 9 to 10 years GMFCS: II to V	Enjoyment	An original questionnaire	45 min, once a week, for 8 months. In total, 30 classes were performed. Aquatic therapy was conducted as a part of the school program and conducted according to the Halliwick concept.	Enjoyment: Levels of enjoyment were high.
Declerck et al. (2016) [21]	RCT	Size: n = 14 Age: 7 to 17 years GMFCS: I to III	Walking ability, Enjoyment	1MWT, 5-point Likert scale	40–50 min, twice a week, for 10 weeks. 5–10 min of warm-up with games and reviewing the tasks learned in the previous session, 20–40 min of learning new tasks, 5–10 min of free play, races, and other games. All participants had an individual program based on decision-making models.	1MWT: Improved significantly more in the intervention group than in the control group (*p* = 0.043). Enjoyment: Levels of enjoyment were high in the intervention group.
Retarekar et al. (2009) [22]	Single case study	Size: n = 1 Age: 5 years GMFCS: III	Walking ability	6MWT, MEEI	40–50 min, 3 times a week, for 12 weeks. 5 min of warm-up, 30–40 min of aerobic exercise program, 5 min of cool down.	The data were analyzed using the 2 SD band method. 6MWT: 27.1% improvement. MEEI: Decreased for some data points.
Fragala-Pinkham et al. (2014) [23]	Single group study	Size: n = 8 Age: 6 to 16 years GMFCS: I to III	Walking ability	6MWT	60 min, twice a week, for 14 weeks. 2–5 min of warm-up, 40–45 min of aerobic exercise, 5–10 min of strength training, 5–10 min of cool down and stretch.	6MWT: Significantly improved from baseline 1 and 2 (*p* = 0.004, 0.001).
Adar et al. (2017) [24]	RCT	Size: n = 32 Age: 4 to 17 years GMFCS: I to IV	HRQOL	Child self-report-PedsQL, parent proxy report-PedsQL	60 min, 5 times a week, for 6 weeks. 10 min of poolside exercises including warming-up, active ROM exercises, and stretching, 50 min of aquatic exercise in the pool. The pool session consisted of 25 min of aerobic exercise, 20 min of active ROM, stretching and strengthening exercises, and 5 min of cool-down.	HRQOL: There were greater improvements in the intervention group than the control group.
Fatorehchy et al. (2019) [25]	Single group study	Size: n = 6 Age: 6 to 10 years GMFCS: I to III	Walking ability	1MWT	50 min, twice a week, for 8 weeks. 10 min of warm up and stretching, 40 min of walking in the pool at different water depths.	1MWT: Significantly improved after the intervention (*p* = 0.041).

GMFCS: Gross Motor Function Classification System; 6MWT: 6-min walk test; MEEI: Modified Energy Expenditure Index; SD; standard deviation; HRQOL: health-related quality of life; 1MWT: 1-min walk test; RCT: randomized controlled trial; PedsQL: Pediatric Quality of Life Inventory; ROM; range of motion.

## Data Availability

The data underlying this article cannot be shared publicly due to privacy concerns of the individuals who participated in the studies. The data will be shared by the corresponding author upon reasonable request.

## Appendix A

**Table A1:** Quality Assessment Tool for Before–After (Pre–Post) Studies with no Control Group.

Author/Year	Outcome	1	2	3	4	5	6	7	8	9	10	11	12	Total
Ogonowska-Slodownik A et al. 2024 [20]	Enjoyment	Yes	No	No	NR	No	Yes	No	No	Yes	No	No	NA	3/12
Retarekar R et al. 2009 [22]	Walking ability	Yes	No	No	NR	No	Yes	Yes	No	Yes	No	Yes	NA	5/12
Fragala-Pinkham MA et al. 2014 [23]	Walking ability	Yes	No	No	NR	NR	No	Yes	Yes	Yes	Yes	Yes	NA	6/12
Fatorehchy S et al. 2019 [25]	Walking ability	Yes	No	No	NR	NR	Yes	Yes	NR	Yes	Yes	No	NA	5/12
NA, not applicable; NR, not reported. Was the study question or objective clearly stated? Were eligibility/selection criteria for the study population prespecified and clearly described? Were the participants in the study representative of those who would be eligible for the test/service/intervention in the general or clinical population of interest? Were all eligible participants that met the prespecified entry criteria enrolled? Was the sample size sufficiently large to provide confidence in the findings? Was the test/service/intervention clearly described and delivered consistently across the study population? Were the outcome measures prespecified, clearly defined, valid, reliable, and assessed consistently across all study participants? Were the people assessing the outcomes blinded to the participants’ exposures/interventions? Was the loss to follow-up after baseline 20% or less? Were those lost to follow-up accounted for in the analysis? Did the statistical methods examine changes in outcome measures from before to after the intervention? Were statistical tests done that provided p values for the pre-to-post changes? Were outcome measures of interest taken multiple times before the intervention and multiple times after the intervention (i.e., did they use an interrupted time-series design)? If the intervention was conducted at a group level (e.g., a whole hospital, a community, etc.) did the statistical analysis take into account the use of individual-level data to determine effects at the group level?														
